# Correction: TP53 copy number expansion is associated with the evolution of increased body size and an enhanced DNA damage response in elephants

**DOI:** 10.7554/eLife.24307

**Published:** 2016-12-20

**Authors:** Michael Sulak, Lindsey Fong, Katelyn Mika, Sravanthi Chigurupati, Lisa Yon, Nigel P Mongan, Richard D Emes, Vincent J Lynch

Sulak M, Fong L, Mika K, Chigurupati S, Yon L, Mongan NP, Emes RD, Lynch VJ. 2016. *TP53* copy number expansion is associated with the evolution of increased body size and an enhanced DNA damage response in elephants. *eLife* **5**:e11994. doi: 10.7554/eLife.11994.

Published 19, September 2016

After publication we became aware of three incorrect citations in the Materials and methods.

The Notung v2.6 citation was incorrectly cited as Notung v2.6 (Wilkie et al., 2013) and Notung v2.6 (Trapnell et al., 2012), it should have cited Chen et al. 2000.

The Proboscidean body size citation was incorrectly cited as ‘Proboscidean body size data were obtained from previously published studies on mammalian body size evolution (Chen et al., 2000)’, it should have cited Evans et al. 2012.

The article has been corrected accordingly.

